# Role of ClpB From *Corynebacterium crenatum* in Thermal Stress and Arginine Fermentation

**DOI:** 10.3389/fmicb.2020.01660

**Published:** 2020-07-17

**Authors:** Mingzhu Huang, Yue Zhao, Lin Feng, Lingfeng Zhu, Li Zhan, Xuelan Chen

**Affiliations:** ^1^Department of Life Science, Jiangxi Normal University, Nanchang, China; ^2^Key Laboratory of Functional Small Organic Molecule of Ministry of Education, Jiangxi Normal University, Nanchang, China

**Keywords:** ClpB, DnaK, *Corynebacterium crenatum*, thermal stress, arginine

## Abstract

ClpB, an ATP-dependent molecular chaperone, is involved in metabolic pathways and plays important roles in microorganisms under stress conditions. Metabolic pathways and stress resistance are important characteristics of industrially -relevant bacteria during fermentation. Nevertheless, ClpB-related observations have been rarely reported in industrially -relevant microorganisms. Herein, we found a homolog of ClpB from *Corynebacterium crenatum*. The amino acid sequence of ClpB was analyzed, and the recombinant ClpB protein was purified and characterized. The full function of ClpB requires DnaK as chaperone protein. For this reason, *dnaK*/*clpB* deletion mutants and the complemented strains were constructed to investigate the role of ClpB. The results showed that DnaK/ClpB is not essential for the survival of *C. crenatum* MT under pH and alcohol stresses. The ClpB-deficient or DnaK-deficient *C. crenatum* mutants showed weakened growth during thermal stress. In addition, the results demonstrated that deletion of the *clpB* gene affected glucose consumption and L-arginine, L-glutamate, and lactate production during fermentation.

## Introduction

Molecular chaperones are essential for homeostasis in living cells. Their fundamental role is to aid proteins in achieving their functional and final conformations (Hartl and Hayer-Hartl, 2002). ClpB is an ATP-dependent molecular chaperone that reactivates and disaggregates aggregated proteins with the DnaK chaperone system (Mogk et al., 2018). Similar to other ATP-dependent molecular chaperones, ClpB forms a hexameric ring structure to mediate protein disaggregation. The mechanism of the ClpB-catalyzed protein disaggregation is the coupling of ATP hydrolysis with the translocation of polypeptide substrates through the central channel of barrel-shaped hexamers (Li et al., 2015).

Proteolysis is essential under conditions where many misfolded and damaged proteins are likely to accumulate, particularly oxidizing environments, non-physiological pH, or elevated temperatures (Raivio, 2018). The survival rate of bacteria decreases tremendously without ClpB when cells are under various stressful conditions, and ClpB is involved in supporting the virulence of some bacterial pathogens (Squires et al., 1991; Athar et al., 2018; Sangpuii et al., 2018). Multiple enzymes of the central carbon metabolism are potential substrates of ClpB. Hence, this protein has regulatory activity in terms of the metabolism. For example, the majority of ClpB-interacting proteins in *Leptospira interrogans* is associated with metabolic pathways, such as the tricarboxylic acid (TCA) cycle, amino acid metabolism, and glycolysis–gluconeogenesis (Joanna et al., 2018). ClpB orthologs have been identified in plants, fungi, bacteria, and protozoa (Ranaweera et al., 2018). Nevertheless, in microbes employed on the industrial scale, the role of ClpB has not been sufficiently investigated. Industrially relevant bacteria also experience various stresses during fermentation (Oide et al., 2015), and studies on metabolic pathways are extremely important for the production of compounds using industrially relevant bacteria. Thus, the relevance of ClpB in industrially relevant bacteria is worth to be investigated in detail.

*Corynebacterium glutamicum*, an industrial bacterium, has a long history in the industrial production of various amino acids (Lee and Wendisch, 2017). *C. glutamicum* mutant strains have been constructed for a number of useful metabolites, such as polyphenols, organic acids, and alcohols (Litsanov et al., 2012; Kallscheuer et al., 2016; Vogt et al., 2016). The *C. glutamicum* genome contains structural genes coding for chaperone systems including *clpC*, *clpB*, *clpX*, *dnaK*, *clpP1*, and *clpP2* (Engels et al., 2004). Some chaperone systems have been studied in *C. glutamicum*. For example, a *clpC*-deficient *C. glutamicum* mutant exhibited higher levels of gene expression compared with the wild type (Engels et al., 2004). The expression of *clpC* was clearly upregulated at pH 6.0 (Peng et al., 2019). However, most chaperone systems have not been studied in *C. glutamicum*. *Corynebacterium crenatum* is a close relative of *C. glutamicum*, and its mutant strains can also produce various industrial compounds (Su et al., 2014). In this study, we found a ClpB ortholog (an ATP-dependent molecular chaperone) from *C. crenatum*. The *clpB* gene was cloned and heterologously expressed in *Escherichia coli*, and the C-terminal His-tagged fusion protein was purified and characterized. We constructed *dnaK*/*clpB* deletion mutants and the complemented strains to investigate the role of ClpB and DnaK in *C. crenatum* MT (mutant strain that is auxotrophic for biotin and produces L-arginine, laboratory stock). Our investigation demonstrated that ClpB and DnaK are not essential for the survival of *C. crenatum* MT under pH and alcohol stresses. However, *dnaK*/*clpB* deletion mutants showed weakened growth during thermal stress. In addition, we showed that the deletion of the *clpB* gene affected glucose consumption and L-arginine, L-glutamate, and lactate production during fermentation.

## Materials and Methods

### Bacterial Strains, Plasmids, and Growth Conditions

All strains and plasmids used in this study are listed in Table 1. *C. crenatum* strains were grown in Luria–Bertani (LB) medium (containing per liter of distilled water: 10 g of tryptone, 10 g of NaCl, 5 g of yeast extract, pH 7.0) at 30°C. *E. coli* DH5α/BL21 (DE3) strains were cultured in LB medium at 37°C. When required, kanamycin and chloromycetin (Solarbio, China) were used at a concentration of 12.5 μg/ml for *C. crenatum* and 25 μg/ml for *E. coli*. BHI medium (Land bridge, China) was used for the transformation of *C. crenatum* via electroporation. Sucrose medium was used to remove pK18*mobsacB* containing *sacB*, which is a sucrose lethal gene (Chen et al., 2015). The fermentation medium (per liter) was composed of 120 g of glucose, 40 g of corn steep liquor, 1.5 g of KH_2_PO_4_, 20 g of (NH_4_)_2_SO_4_, 0.5 g of MgSO_4_⋅7H_2_O, 0.02 g of FeSO_4_⋅7H_2_O, 0.05 g of MnSO_4_⋅2H_2_O, 30 g of CaCO_3_, 5 × 10^–4^ g of thiamine, 8 × 10^–5^ g of biotin, and pH 7.0. The seed medium (per liter) of *C. crenatum* consisted of 40 g of (NH_4_)_2_SO_4_, 20 g of corn steep liquor, 1.5 g of urea, 30 g of glucose, 1 g of KH_2_PO_4_, 0.5 g of MgSO_4_⋅7H_2_O, and pH 7.0 (Chen et al., 2015). Growth analyses were performed using the defined medium CGXII (containing per liter of distilled water: 20 g of (NH_4_)_2_SO_4_, 40 g of glucose, 5 g of urea, 1 g of K_2_HPO_4_, 1 g of KH_2_PO_4_, 0.25 g of MgSO_4_⋅7H_2_O, 10 mg of MnSO_4_⋅H_2_O, 10 mg of FeSO_4_⋅7H_2_O, 0.25 mg of CaCl_2_⋅2H_2_O, 0.2 mg of CuSO_4_⋅5H_2_O, 1 mg of ZnSO_4_⋅7H_2_O, 0.02 mg of NiCl_2_⋅6H_2_O, 0.2 mg of biotin, 40 g of 3-(*N*-morpholino) propanesulfonic acid, pH 7.0).

**TABLE 1:** Strains and plasmids in this study.

**Strains/plasmids**	**Characteristics**	**Resources**
**Plasmids**		
pK18*mobsacB*	Mobilizable vector, allows for selection of double crossover in *C. crenatum*, Km^R^, *sacB*	Biovector
pXMJ19	Shuttle vector for overexpression, Chl^R^	Biovector
pK18*mobsacB*-*clpB*	A derivative of pK18*mobsacB*, harboring *clpB* fragments	This work
pK18*mobsacB*-*dnaK*	A derivative of pK18*mobsacB*, harboring *dnaK* fragments	This work
pXMJ19- *clpB*	A derivative of pXMJ19, harboring *clpB* gene fragments	This work
pXMJ19- *dnaK*	A derivative of pXMJ19, harboring *dnaK* gene fragments	This work
**Strains**		
*E. coli* DH5α	Clone host strain	Invitrogen
*E. coli* BL21(DE3)	Recombinant expression	Invitrogen
*E. coli* BL21 (DE3)-*clpB*	Harboring pXMJ19- *clpB*	This work
*C. crenatum* MT	mutation strain with auxotrophy for biotin, and producing L-Arg	Lab Stock (Chen et al., 2015)
*C. crenatum*Δ*clpB*	*C. crenatum* MT with a deletion of the *clpB* gene	This work
*C. crenatum*Δ*dnaK*	*C. crenatum* MT with a deletion of the *dnaK* gene	This work
*C. crenatum*Δ*dnaK*Δ*clpB*	*C. crenatum* MT with deletion of the *dnaK* and *clpB* genes	This work
*C. crenatum*Δ*clpB*(pXMJ19*-clpB*)	Complemented strain of *C. crenatum*Δ*clpB*	This work
*C. crenatum*Δ*dnaK*(pXMJ19*-dnaK*)	Complemented strain of *C. crenatum*Δ*dnaK*	This work
*C. crenatum* (pXMJ19)	*C. crenatum* MT harboring pXMJ19	This work

### Construction of Plasmids and Strains

The primers applied in this study are shown in Table 2. The *clpB* and *dnaK* genes in *C. crenatum* MT were deleted using pK18*mobsacB* (Schafer et al., 1994). In brief, we amplified upstream and downstream sequences of *clpB* and *dnaK* by specific primers. The upstream and downstream sequences were fused using overlapping PCR. The fused fragments were ligated into pK18mobsacB by NovoRec^®^Plus PRC kit (Novoprotein, China). The recombinant plasmids were used for the transformation of *C. crenatum* MT using electroporation as previously reported (Zhang et al., 2017). The single exchange clones were selected using kanamycin, whereas the double crossover clones were checked using PCR. The coding sequences of *clpB* and *dnaK* were amplified by the primers with His-tag at the C-terminus. The PCR-amplified *clpB* and *dnaK* were ligated into the *E. coli*–*C. glutamicum* shuttle vector pXMJ19. The recombinant vectors were used for the transformation of *C. crenatum* Δ*clpB* and *C. crenatum* Δ*dnaK* as complemented strains, respectively. The plasmid was used for the transformation of *E. coli* BL21 (DE3) via heat shock. The *clpB* gene was expressed in *E. coli* BL21 (DE3), and the ClpB protein was purified as a hexamer as previously reported (Lupoli et al., 2016; Rizo et al., 2019). In brief, *E. coli* BL21 (DE3)-*clpB* was incubated in LB medium for heterologous gene expression at 37°C, supplemented with 0.5 mM isopropyl-β-D-thiogalactoside, and cultivated for 12 h. The proteins were harvested and analyzed by SDS-PAGE (Wang et al., 2020). Whole cells were disrupted using sonication (Wang et al., 2020). Subsequently, the recombinant protein was purified using Ni-NTA affinity chromatography (Sangon Biotech, China). Total protein content was determined using a BCA protein assay kit (Nanjing Jiancheng, China).

**TABLE 2:** Primers and sequences in this study.

**Primers**	**Primer sequences (5′-3′)**	**Target**
*clpB*-up-F	AACGACGGCCAGTGCCAAGCTCAATGAGTATGGCGTGGCGTA	Upstream fragment of *clpB*
*clpB* -up-R	CAATGACAGGATCGAAACGTACTCATCGCCTAACTC	Upstream fragment of *clpB*
*clpB*-down-F	GATGAGTACGTTTCGATCCTGTCATTGGCCGTGAC	Downstream fragment of *clpB*
*clpB*-down-R	CGGTACCCGGGGATCCTCTAGCGTTCCTTGGAAGCTGCATCG	Downstream fragment of *clpB*
*dnaK*-up-F	AACGACGGCCAGTGCCAAGCTGACGTGCAGTAGGAATTGACCTTGG	Upstream fragment of *dnaK*
*dnaK*-up-R	ATGCGCTGGAACTCTGCACGGGTCAACGATACGCTGATCCCAGTCGT	Upstream fragment of *dnaK*
*dnaK*-down-F	ATCAGCGTATCGTTGACCCGTGCAGAGTTCCAGCGCATCAC	Downstream fragment of *dnaK*
*dnaK*-down-R	CGGTACCCGGGGATCCTCTAGGACAGACCGGAGCCGTCCTGAATG	Downstream fragment of *dnaK*
*clpB*-F	AAACAGAATTAATTAAGCTTATGAGTTCATTCAATCCAACTACCA	ORF of *clpB*
*clpB*-R	GTACCCGGGGATCCTCTAGATTAATGATGATGATGATGATGATGGACCG CCTTGGAAACGTCGAGCTTC	ORF of *clpB*
*dnaK*-F	AACAGAATTAATTAAGCTTATGGGACGTGCAGTAGGAATT	ORF of *dnaK*
*dnaK*-R	TTAATGATGATGATGATGATGATGCTTCTTATCCTCACCATTGTC	ORF of *dnaK*
pXMJ19 check-F	CGGCTCGTATAATGTGTGGA	Recombinant pXMJ19 vector detecting
pXMJ19 check-R	ATCTTCTCTCATCCGCCAAA	Recombinant pXMJ19 vector detecting
M13-F	CGCCAGGGTTTTCCCAGTCACGAC	Recombinant pK18*mobsacB* vector detecting
M13-R	GAGGGGATAACAATTTCACACAGG	Recombinant pK18*mobsacB* vector detecting

### Cell Growth Analyses

For liquid growth experiments, the strains were inoculated in LB medium for approximately 18 h and were subsequently transferred into 100 ml of fresh CGXII medium to an initial OD at 562 nm of 0.06. Approximately 3 ml of each culture was collected every hour for OD measurement. For solid growth experiments, the strains were inoculated in LB medium for approximately 18 h. Subsequently, 1 ml of the medium was transferred into 50 ml of fresh LB medium. When an OD of 1 was reached, the cultures were diluted using phosphate-buffered saline containing 10 mM Na_2_HPO_4_, 137 mM NaCl, 2.7 mM KCl, and 2 mM KH_2_PO_4_, pH 7.4 (10^–2^–10^–6^). Approximately 5 μl of the dilutions was spot-plated on CGXII solid media (Mashruwala et al., 2019). Cultures of complemented strains were grown in medium with additional chloromycetin and IPTG.

### ATPase Test

The fusion ClpB protein was incubated with buffer A (10 mM MgCl_2_, 1 mM dithiothreitol, BSA 0.1 mg/ml, 25 mM Tris, 0.05–2.5 mM ATP; pH 7.6) for 10 min at 30°C in accordance with Kajfasz and Santagata’s method (Santagata et al., 1999; Kajfasz et al., 2009) with slight modifications. EDTA (25 mM) was added to stop the catalytic reaction, and the reaction buffer was frozen at −80°C. The release of inorganic phosphate from ATP was measured using a Micro Tissue Inorganic Phosphorus Content assay kit (Solarbio, China) following the manufacturer’s protocol.

### Fermentation in Shake Flasks

The strains were activated for 24 h on LB solid media. The activated strains were cultured in a seed medium for 18 h at 30°C with agitation at 200 rpm. Approximately 2 ml of each seed culture was transferred into 25 ml of fermentation medium. The fermentations were incubated at 30°C and 200 rpm, and 200 μl of cultures was collected every 12 h. Glucose concentration was determined by 3,5-dinitrosalicylic acid colorimetry (Chen et al., 2015). The concentrations of L-arginine were determined with a Sykam S-433D amino acid analyzer (Sykam Co., Ltd., Germany) (Chen et al., 2015). L-glutamate and lactate were measured enzymatically using a bioanalyzer (SBA-40E, Shandong, China). Cell growth was monitored by measuring the OD_562_ (1 OD_562_ = 0.375 g/L dry cell weight) with a spectrophotometer (BG-XM496, Beijing, China) after dissolving CaCO_3_ with HCl.

### Bioinformatics and Statistical Datum Analysis

The 3D structure model of ClpB was constructed through protein alignment with SWISS-MODEL (Bienert et al., 2017; Bertoni et al., 2017). The differences among groups were determined by ANOVA. All statistical data were analyzed using GraphPad Prism 5. All experiments were conducted in triplicate, and all data are presented as mean ± standard deviation.

## Results and Discussion

### Analysis of the ClpB Sequence

**FIGURE 1:**
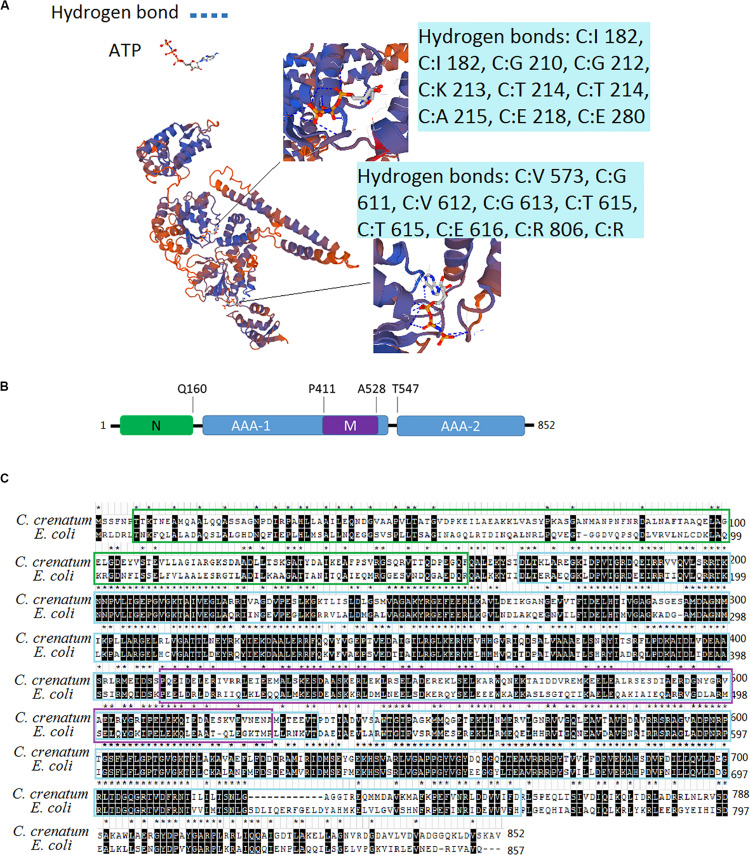
Analysis of the ClpB sequence from *C. crenatum*. **(A)** 3D structure model of ClpB. The enlarged section shows ATP hydrolysis domains, and the blue boxes show amino acid residues forming hydrogen bonds. **(B)** Domain organization of ClpB. ClpB consists of an N-terminal (N) domain, two AAA domains (AAA-1 and AAA-2), and an inserted middle (M) domain. **(C)** Multiple sequence alignment. Identical residues are marked with stars and black background; characters in green box represent N-terminal domain; characters in blue box represent AAA domains; characters in purple box represent middle domain.

We obtained the DNA sequence from the whole genome shotgun sequence of *C. crenatum* (National Center of Biotechnology Information, NCBI Reference Sequence: GCA_000380545.1). The 3D structure model of ClpB was constructed on the basis of the sequences of ClpB proteins and the crystal structure of ClpB from *Mycobacterium tuberculosis* (Figure 1A). ClpB belongs to Hsp100 or the Clp family (caseinolytic protease). A distinct characteristic of Hsp100 is the presence of ATP hydrolysis domains (AAA domain) (Baker and Sauer, 2006). ClpB of *C. crenatum* MT contains two conserved ATP hydrolysis domains, AAA1 and AAA2, forming 12 and 10 hydrogen bonds, respectively. AAA1 and AAA2 provide ClpB with the ability of ATP hydrolysis required for restructuring substrates (Baker and Sauer, 2006). Conserved substrate-binding “pore loops” in the ATP hydrolysis domains contain essential tyrosine residues that mechanically couple ATP hydrolysis to translocation (Tessarz et al., 2008). Besides the two ATP hydrolysis domains, the domains of ClpB (Figure 1B) include an N-terminal domain (N) and a coiled-coil middle domain (M). The N-terminal domain provides ClpB with the ability to bind substrates (Gates et al., 2017). The middle region within the first AAA cassette contains a bundle of four helices approximately 120 amino acids long, which is required for ClpB interactions with DnaK and mediates allosteric functions during disaggregation and hydrolysis (Haslberger et al., 2007). The two sequence alignments indicated that the protein sequences of ClpB between *C. crenatum* MT and *E. coli* are highly conserved in the two ATP hydrolysis domains (Figure 1C), in which all 15 amino acid residues forming hydrogen bonds are conserved. The N-terminal region showed low similarity, which is loosely associated with the core domain and is not necessary for ClpB activity (Nagy et al., 2006).

### ATPase Activity

**FIGURE 2:**
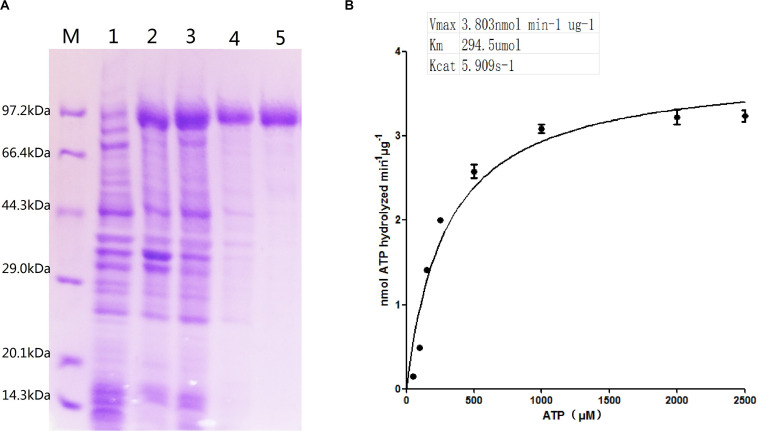
Characterization of ATPase activity. **(A)** Expression of the *clpB* gene and purification of ClpB; overexpressed recombinant ClpB (lanes 2–3), control (lane 1), samples of wash buffer eluent (lane 4), and purified ClpB (lane 5). **(B)** Michaelis–Menten equation of ClpB.

The recombinant ClpB protein with His-tag at the C-terminus was purified from *E. coli* BL21 (DE3) -ClpB by using Ni-NTA affinity resin (Figure 2A). Compared with the control (lane 1), an apparent ∼93 kDa protein band in lanes 2–3 was visualized, demonstrating that the *clpB* gene was successfully expressed. A single protein band (lane 5) showed successfully purified recombinant ClpB. For the measurement of ClpB activity, 20 μg of recombinant ClpB protein was incubated with 1 ml of buffer A at 30°C for 10 min. The Michaelis–Menten equation of ClpB shown in Figure 2B revealed that ClpB hydrolyzed ATP with V_max_ = 3.803 nmol min^–1^ μg^–1^ and *K*_m_ = 294.5 μmol, corresponding to 1 molecule of ClpB hydrolyzing 5.909 molecules of ATP in 1 s.

**FIGURE 3:**
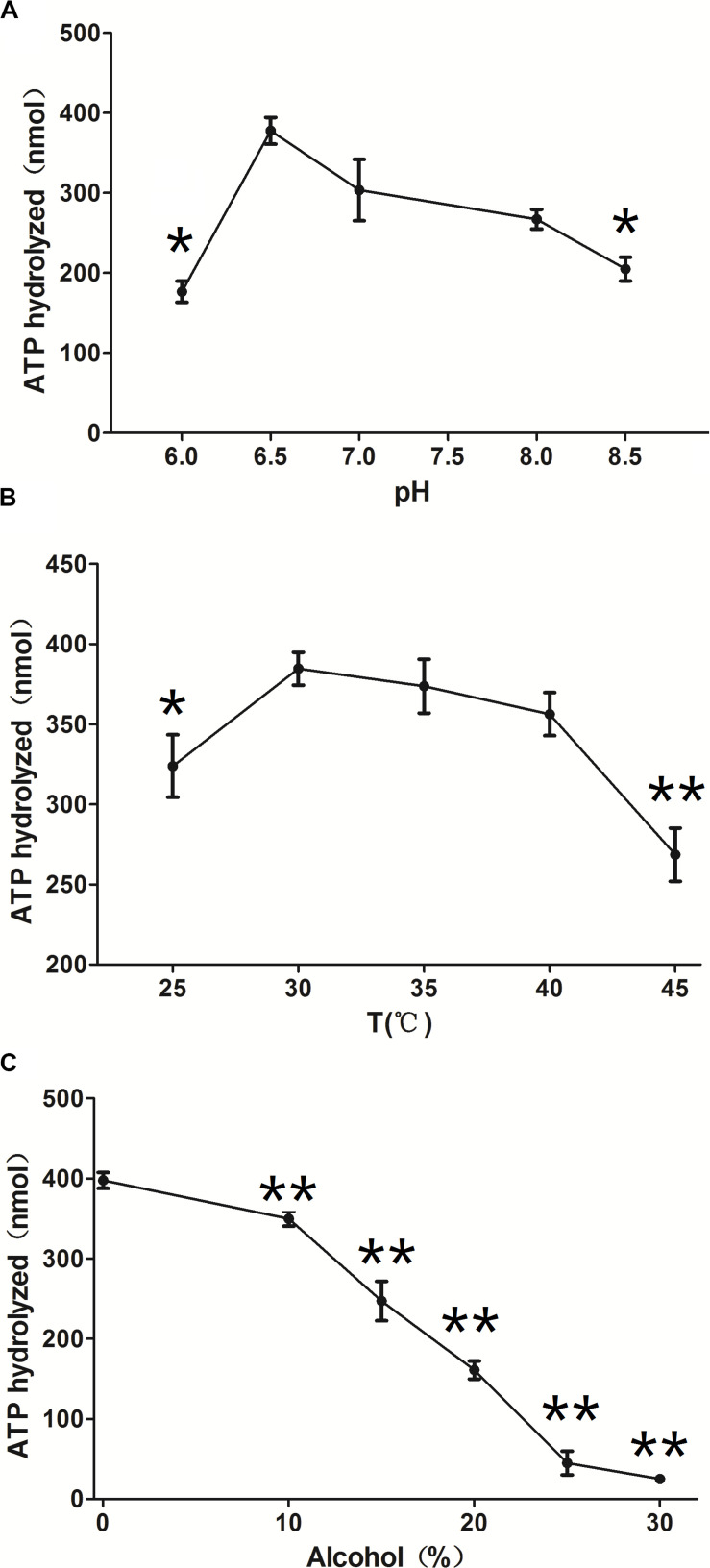
Effects of different conditions on ClpB activity. **(A)** pH. The reactions were performed at 30°C at different pH values (6.0, 6.5, 7.0, 8.0, and 8.5). **(B)** Temperature. The reactions were performed at different temperatures (25, 30, 35, 40, and 45°C). **(C)** Ethanol. The reactions were performed at different ethanol concentrations (0, 10, 15, 20, 25, and 30%). Compared with the control group (pH 7.0, temperature 30°C, and 0% alcohol), differences were considered statistically significant at *p* < 0.05. “**” indicates *p* < 0.01; “*” indicates 0.01 < *p* < 0.05.

ClpB is involved in the response to thermal, oxidative, osmotic, pH, ethanol, and starvation stresses of microorganisms and protects vital cellular proteins encountering stress conditions (Meibom et al., 2008; Pan et al., 2012; Krajewska et al., 2017; Tripathi et al., 2020). Therefore, we measured the ATPase activity of ClpB under different conditions to reflect different types of stresses. Different pH affects the stability of all cellular proteins and their function. *C. glutamicum*, a close relative of the here investigated *C. crenatum*, is an acid-sensitive strain (Liu et al., 2016). However, Figure 3A shows that the groups of pH = 9.0 and 8.5 were significantly reduced compared with the group of pH = 7.0, and the optimal pH of ClpB was 6.5. This finding indicates that ClpB is still able to maintain high activity for the homeostasis of cellular proteins in acidic conditions. Athar et al. (2018) also certified that ClpB performs important functions during low-pH stress response. Many previous observations showed that ClpB contributes to the survival of bacteria during heat stress (Squires et al., 1991; Athar et al., 2018; Sangpuii et al., 2018). Our results showed (Figure 3B) that the activity of ClpB was relatively unaltered at 30–40°C and decreased evidently at 25 and 45°C. ATPase activity decreased by only 7% at 40°C relative to the highest value (at 30°C), which revealed that ClpB efficiently functioned during heat stress, but not during cold stress. Improving resistance to alcohols is an important aspect in engineering strains for producing alcohols (Borden and Papoutsakis, 2007). Hence, the ATPase activity of ClpB was determined in presence of different ethanol concentrations (Figure 3C). Figure 3C indicates that the activity of ClpB was unstable under alcohol conditions, and ClpB is probably useless in *C. crenatum* MT under high alcohol concentrations.

### Inactivation of ClpB Confers a Growth Defect Under Thermal Stress

**FIGURE 4:**
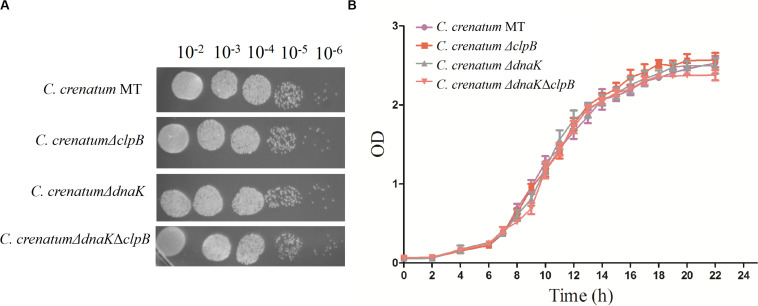
Effects of *clpB* and *dnaK* deletion on the growth rate at 30°C. Solid growth analysis **(A)**: cultures of the strains were grown in liquid medium at 30°C. At OD_562_ = 1, the cultures were diluted by 10^– 2^-fold to 10^– 6^-fold, and 5 μl of each dilution was spotted on CGXII plates. The plates were incubated at 30 C. Liquid growth analysis **(B)**: strains were cultured in LB medium for 18 h at 30°C and subsequently inoculated into 50 ml of fresh CGXII at 30°C.

**FIGURE 5:**
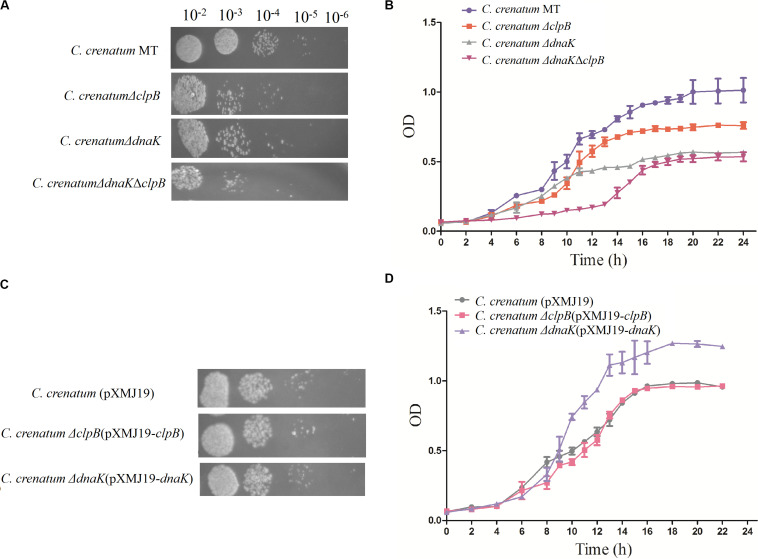
Effects of *clpB* and *dnaK* deletion on the growth rate at 40°C. Solid growth analysis **(A,C)**: cultures of the strains were grown in liquid medium at 30°C. At OD_562_ = 1, the cultures were diluted by 10^– 2^-fold to 10^– 6^-fold, and 5 μl of each dilution was spotted on CGXII plates. The plates were incubated at 40°C. Liquid growth analysis **(B,D)**: strains were cultured in LB medium for 18 h at 30°C and subsequently inoculated into 50 ml of fresh CGXII at 40°C. Cultures of complemented strains were grown in medium with additional chloromycetin and IPTG.

ClpB plays a vital role in the survival of microorganisms during stressful conditions (Squires et al., 1991; Athar et al., 2018; Sangpuii et al., 2018). In general, bacterial ClpB cooperates with DnaK cochaperone to refold and rescue aggregated proteins, thereby helping cells survive under stressful conditions (Lupoli et al., 2016). Therefore, we constructed *dnaK*/*clpB* deletion mutants and complemented strains to investigate the role of ClpB and DnaK in stress. However, our study demonstrated that ClpB and DnaK from *C. crenatum* MT are not essential for the survival of *C. crenatum* MT under alcohol and pH stress (Supplementary Figures S1, S2). The cooperation of ClpB and chaperones in substrate disaggregation is species-specific (Schlee et al., 2004; DeSantis and Shorter, 2012; Ngansop et al., 2013). The M domain of ClpB corresponds to the species-specific cooperation with chaperones (Miot et al., 2011). The two-sequence alignment showed that the sequence identity between *C. crenatum* MT and *E. coli* within the middle domain of ClpB was only ∼42% (Figure 1C), which indicated species-specific function. In general, industrial fermentation is carried out below 35°C. These processes demand large quantities of water to cool the heat generated by the metabolism of microbes, particularly during hot seasons and in tropical regions (Oide et al., 2015). Thus, studying the thermal stress tolerance of microorganisms reduces the costs of maintaining optimal fermentation temperature (Oide et al., 2015). We first determined whether the growth of *C. crenatum* Δ*clpB*, *C. crenatum* Δ*dnaK*, and *C. crenatum* Δ*dnaK*Δ*clpB* differ from that of *C. crenatum* MT at an optimal temperature. Thus, the strains were cultured for solid and liquid growth analyses at 30°C (Figures 4A,B). The growth of *C. crenatum* Δ*clpB*, *C. crenatum*Δ*dnaK*, and *C. crenatum* Δ*dnaK*Δ*clpB* showed no significant difference compared with that of *C. crenatum* MT. The number of colonies of *C. crenatum*Δ*clpB*, *C. crenatum*Δ*dnaK*, and *C. crenatum*Δ*dnaK*Δ*clpB* was smaller than that of *C. crenatum* at 40°C (Figure 5A). The differences of growth were obvious through liquid growth analyses (Figure 5B), and *C. crenatum* Δ*dnaK* and *C. crenatum*Δ*dnaK*Δ*clpB* showed the weakest growth rate, indicating that ClpB and DnaK contribute to thermal stress tolerance. Compared with the wild type of *Salmonella typhimurium*, Δ*clpB* strains did not show any sensitivity to a temperature of 37°C but were hypersusceptible (*p* < 0.001) to a temperature of 42°C (Sangpuii et al., 2018). When subjected to high temperature (50°C), the survival of the Δ*clpB* mutant of *Francisella tularensis* was compromised, whereas the CFU of the wild-type strains did not drop significantly (Athar et al., 2018). Thermal stress results in the massive misfolding of proteins and the aggregation of proteins, which is toxic for bacteria. Cell survival during heat stress requires the reactivation of aggregated proteins by ClpB and DnaK, which indicates the essential role of ClpB and DnaK in thermotolerance (Weibezahn et al., 2004; Acebrón et al., 2008). Furthermore, the growth rate under thermal stress was observed through the complemented strains (Figures 5C,D). The deficient growth rate was restored in *C. crenatum* Δ*clpB* (pXMJ19-*clpB*). Unexpectedly, the growth rate of *C. crenatum* Δ*dnaK* (pXMJ19-*dnaK*) was significantly increased compared with that of *C. crenatum* (pXMJ19). These results suggested that the DnaK/ClpB chaperone system plays an important role in thermotolerance, but DnaK possesses a broader function with other cochaperones. DnaK also collaborates with GrpE and DnaJ to prevent protein aggregation and solubilize protein aggregates, which is critical in the resistance to thermal stress (Acebrón et al., 2008).

### Fermentation in Shake Flasks

**FIGURE 6:**
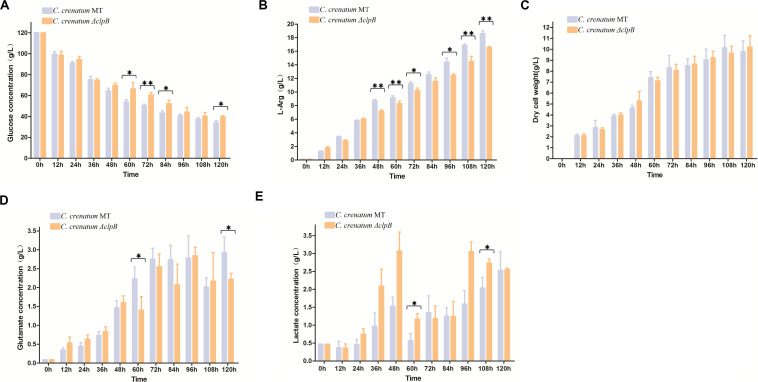
Fermentation of *C. crenatum* MT and *C. crenatum* Δ*clpB*. **(A)** Glucose consumption. **(B)** L-arginine production. **(C)** Dry cell weight. **(D)** L-glutamate production. **(E)** Lactate production. Differences were considered statistically significant at *p* < 0.05. “**” indicates *p* < 0.01; “*” indicates 0.01 < *p* < 0.05. Initial concentrations of 0.47 g/L lactate, 0.11 g/L L-arginine and 0.087 g/L L-glutamate are due to the supplementation of corn steep liquor to the cultivation medium.

**FIGURE 7:**
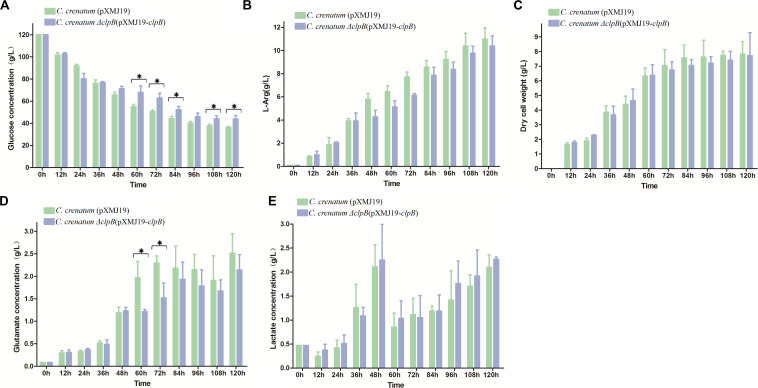
Fermentation of *C. crenatum* Δ*clpB* (pXMJ19-*clpB*) and *C. crenatum* (pXMJ19). **(A)** Glucose consumption. **(B)**, L-arginine production. **(C)** Dry cell weight. **(D)** L-glutamate production. **(E)** Lactate production. Differences were considered statistically significant at *p* < 0.05. “*” indicates 0.01 < *p* < 0.05. Initial concentrations of 0.47 g/L lactate, 0.11 g/L L-arginine and 0.087 g/L L-glutamate are due to the supplementation of corn steep liquor to the cultivation medium.

Various valuable chemical compounds have been produced using glucose through fermentation. Therefore, the understanding of glucose metabolism is essential for industrially relevant bacteria (Sekine et al., 2001). *C. crenatum* Δ*clpB* displayed relatively lower glucose consumption and L-arginine production than *C. crenatum* MT during the late period of fermentation (Figures 6A,B). L-arginine is biosynthesized from L-glutamate through citrulline and ornithine in cellular metabolic pathways (Chen et al., 2015). Figure 6C shows that *clpB* deletion affected the L-glutamate production of *C. crenatum* MT, especially at 60 and 120 h. Lactate is one of the major organic acids excreted to the broth during fermentation (Xu et al., 2009). At the early phase of fermentation, the concentration of lactate showed net consumption, which may be caused by low-density cells and pure medium containing a certain amount of lactic acid (0.47 g/L). The accumulation of lactate was significantly increased in *C. crenatum* Δ*clpB* strain at 60 and 108 h (Figure 6D). Reduction of glutamate and increase of lactic acid may be the reasons for the decrease of arginine production. As a complement, the fermentation of *C. crenatum* Δ*clpB* (pXMJ19-*clpB*) and *C. crenatum* (pXMJ19) was performed, and the cultures were collected and tested. Deletion of *clpB* did not affect the dry cell weight of *C. crenatum* during fermentation (Figures 6C, 7C). Figure 7 shows that the deficient L-arginine production was restored in *C. crenatum* Δ*clpB* (pXMJ19-*clpB*). The glucose consumption of *C. crenatum* Δ*clpB* (pXMJ19-*clpB*) was significantly increased compared with that of *C. crenatum* (pXMJ19); however, L-glutamate production decreased due to the overexpression of the *clpB* gene. The strains might encounter a disadvantageous circumstance with the accumulation of toxic metabolites (Wu et al., 2016) and consumption of nutrition at the late period of fermentation (108–120 h), resulting in massive protein misfolding. The interactional proteins of ClpB include enzymes for major metabolic pathways, such as glycolysis–gluconeogenesis, TCA cycle, and amino acid metabolism (Joanna et al., 2018). The metabolism of *C. crenatum* Δ*clpB* may be subjected to protein misfolding.

## Conclusion

We found a homolog of ClpB from *C. crenatum* MT. The amino acid sequence of ClpB was analyzed, and the recombinant ClpB protein was purified and characterized. The full function of ClpB requires DnaK as chaperone protein. For this reason, *dnaK*/*clpB* deletion mutants and complemented strains were constructed to investigate the role of ClpB. Our investigation demonstrated that ClpB is not essential for the survival of *C. crenatum* MT under pH and alcohol stresses. The growth of *C. crenatum*Δ*clpB*, *C. crenatum*Δ*dnaK*, and *C. crenatum*Δ*dnaK*Δ*clpB* showed no significant difference compared with that of *C. crenatum* MT at optimal temperature. However, growth test under thermal stress indicated that ClpB and DnaK contribute to thermal stress tolerance. Deletion of *clpB* affected glucose consumption and L-glutamate, lactate, and L-arginine production during fermentation. The strains encountered a disadvantageous circumstance with the accumulation of toxic metabolites and consumption of nutrition at the late period of fermentation (108–120 h), which might lead to protein aggregation, and the metabolism of *C. crenatum*Δ*clpB* may be subjected to protein misfolding. The underlying mechanism behind the ClpB protein function remains unknown, and the ClpB of industrially relevant bacteria must be further investigated.

## Data Availability Statement

The datasets generated for this study can be found in the National Center of Biotechnology Information/NZ_AQPS01000020.1/https://www.ncbi.nlm.nih. gov/nuccore/NZ_AQPS01000020.1.

## Author Contributions

MH and XC contributed to research work and manuscript writing. MH contributed to construction of plasmids and strains and bioinformatics analysis. YZ and LF contributed to fermentation. LZhu and LZha contributed to the testing of ATPase and cell growth. All authors contributed to the article and approved the submitted version.

## Conflict of Interest

The authors declare that the research was conducted in the absence of any commercial or financial relationships that could be construed as a potential conflict of interest.
