# Supplementary material for: Role of ClpB From Corynebacterium crenatum in Thermal Stress and Arginine Fermentation
Source: Front Microbiol. 2020 Jul 17;11:1660. doi: 10.3389/fmicb.2020.01660 (PMC7380099; doi:10.3389/fmicb.2020.01660)
Supplement: Supplementary file 1 [file Table_1.DOCX]

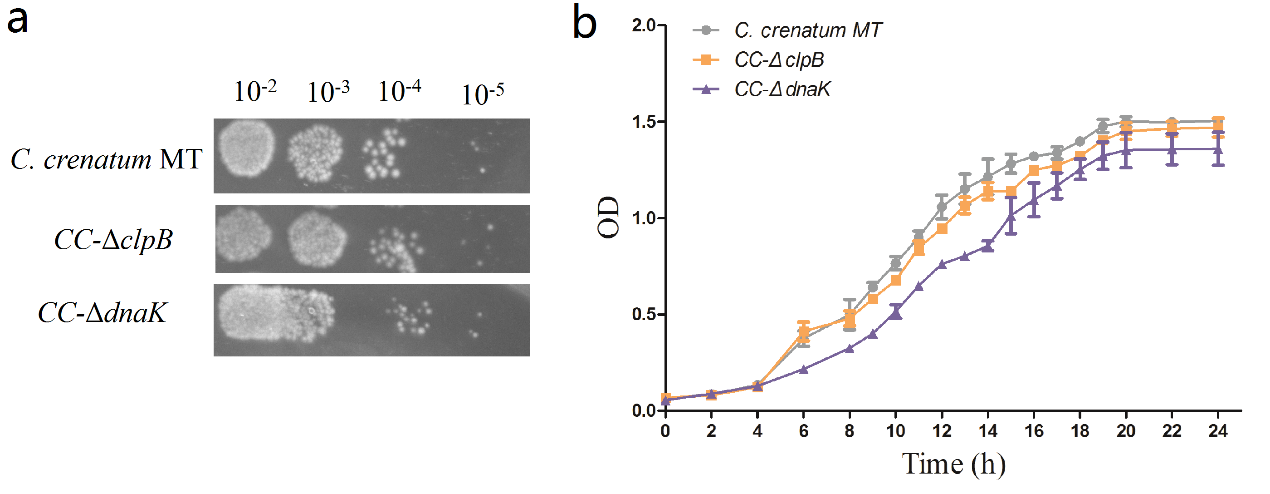


Figure S1. Effects of *clpB* and *dnaK* deletion to alcohol tolerance. (a) Solid growth analysis: cultures of the strains were grown in liquid medium at 30 °C, At OD562=1, the cultures were diluted by 10^−2^-fold to 10^−6^-fold, and 5 μl of each dilution was spotted on CGXII plates with 5% ethanol. The plates were incubated at 30 °C. (b) Liquid growth analysis: strains were cultured in LB for 18 h at 30 °C and subsequently inoculated into 50 ml of fresh CGXII with 5% ethanol at 30 °C.


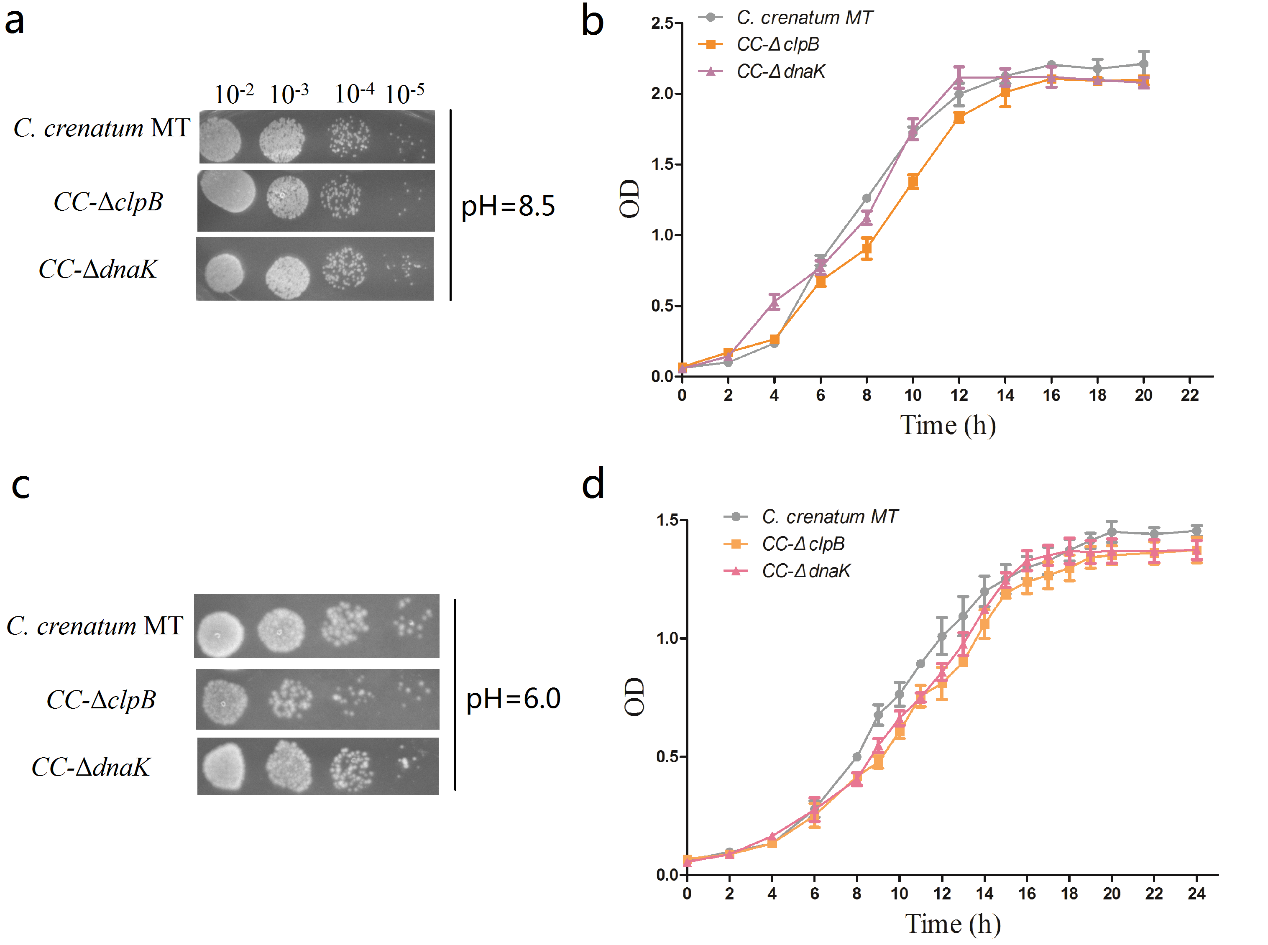


Figure S2. Inactivation of *clpB* and *dnaK* influenced the growth rate in medium with initial pH=6.0 or 8.5. Solid growth analysis (a and c), cultures of all the strains were grown in LB liquid medium at 30 ℃. At OD562=1, the cultures were diluted by 10−2-, 10−3-, 10−4-, 10−5-, and 10−6-fold, and 5 μL of each dilution was spotted on CGXII plates (pH=6.0 or 8.5). The plates were incubated at 30 ℃. Liquid growth analysis (b and d), strains were cultured in LB at approximately 18 h at 30 ℃ and subsequently inoculated into 50 mL fresh CGXII (pH=6.0 or 8.5) at 30 ℃.
